# A framework for Surgical Quality Assurance (SQA) in randomized controlled trials in gastrointestinal surgery: an international Delphi consensus study

**DOI:** 10.1016/j.eclinm.2025.103634

**Published:** 2025-11-13

**Authors:** Dillen C. van der Aa, Sofie P.G. Henckens, Hendrik Jaap Bonjer, Jurriaan B. Tuynman, Marc G. Besselink, Natalie S. Blencowe, Goerge B. Hanna, Mark I. van Berge Henegouwen, Sheraz R. Markar, Suzanne S. Gisbertz, DELPHI-study group

**Affiliations:** a Amsterdam UMC, Location University of Amsterdam, Department of Surgery, Amsterdam, the Netherlands; b National Institute for Health and Care Research Bristol Biomedical Research Centre, Bristol Centre for Surgical Research, Bristol Medical School, Population Health Sciences, University of Bristol, Bristol, United Kingdom; c St Mary's Hospital, Imperial College, London, United Kingdom; d Department of General Surgery, Oxford University Hospitals NHS Foundation Trust, Oxford, United Kingdom

**Keywords:** Surgical quality assessment, Randomized controlled trials, Quality, Gastrointestinal trials

## Abstract

**Background:**

Randomized controlled trials (RCTs) in gastrointestinal surgery often lack standardized approaches to Surgical Quality Assurance (SQA), which threatens both internal and external validity. The technical complexity and inherent inter-surgeon variability in gastrointestinal procedures pose significant challenges for standardizing interventions and ensuring reproducible outcomes. SQA involves credentialing of surgeons, standardization of surgical techniques, and monitoring of performance, but consensus on essential components has been missing. To address this gap, we conducted a four-round Delphi consensus process with 40 international experts in gastrointestinal surgery and clinical trials.

**Methods:**

Experts from 15 countries were invited to rate the relevance of potential SQA items using a 5-point Likert scale from December 2023 to December 2024. The checklist was structured into three domains: credentialing, standardization of surgical techniques, and performance monitoring. Items rated as 4 (‘important’) or 5 (‘very important’) by at least 80% of experts were included in the final SQA checklist.

**Findings:**

Consensus was reached on 13 essential SQA items including a minimum annual case volume per center, a minimum surgeon case volume (overall and annually), standardized reporting guidelines, pretrial education (written materials and videos), standardization of surgical approach, standardized extent of lymphadenectomy, proctoring surgeons (without and with limited experience), periodic pathological assessment, and performance monitoring through Case Report Forms or patient file data.

**Interpretation:**

This Delphi study establishes an international expert consensus framework of 13 items for Surgical Quality Assurance in RCTs in gastrointestinal surgery. This framework is expected to be widely utilized to enhance the quality of surgical trials by improving the internal and external validity. Adoption of these standards may help strengthen the credibility, comparability, and clinical relevance of future surgical RCTs.

**Funding:**

This study received no funding.

**Research in context**

**Evidence before this study**

Although several publications highlight the impact of surgical variability on outcomes in surgical randomized controlled trials (RCTs), we identified no prior studies that proposed a structured, consensus-based framework specifically for Surgical Quality Assurance (SQA) in gastrointestinal cancer surgery trials. Most studies focus on single domains (e.g. credentialing or technique standardization) or individual trial protocols, but no internationally validated checklist existed across the three key domains of SQA: credentialing, standardization, and performance monitoring.

**Added value of this study**

This is the first international Delphi consensus study to define core components of SQA in randomized controlled trials in gastrointestinal cancer surgery. Forty-four experts across 15 countries participated in a four-round Delphi process, resulting in a 13-item checklist that spans credentialing of surgeons and centers, standardization of surgical techniques (including lymphadenectomy and surgical approach), and monitoring of performance during trials. The checklist reflects real-world feasibility and was shaped by expert feedback, including considerations of practicality and global applicability. This structured framework addresses a long-standing gap in surgical trial methodology.

**Implications of all the available evidence**

The adoption of this 13-item framework may improve the internal and external validity of surgical RCTs by reducing performance bias, enhancing reproducibility, and enabling meaningful cross-study comparisons. The checklist offers researchers a practical tool to implement SQA measures in upcoming trials, and its retrospective application to published studies may also support critical appraisal of trial quality.

## Introduction

The lack of consensus-based standards for Surgical Quality Assurance (SQA) affects both the internal and external validity of randomized controlled trials (RCTs) in gastrointestinal (GI) surgery. SQA aims to standardize interventions by standardization of surgical techniques, credentialing of surgeons and monitoring of performances during trials. The technical complexity of surgery poses a significant challenge in designing surgical RCTs, which often involve multiple centers with variability in surgical performance across surgeons, institutions, and countries. Since no two surgical procedures are identical, it is difficult to establish a subsequent evaluation of clinical outcomes.^1^

A systematic review of multicenter RCTs for gastro-esophageal cancer revealed significant variability in study design and SQA, with fewer than 30% of 80 surgical RCTs standardizing interventions and adherence monitoring conducted in only 28%.^2^ Additionally, intervention reporting remains inadequate, particularly regarding materials used, such as staff training and patient education resources in surgical RCTs. Furthermore, fewer than 6% of 54 surgical trials reported how clustering was accounted for in statistics or sample size calculations.^3^^,^^4^ Lack of standardization in surgical techniques can blur differences between study groups, introduce performance bias, and hinder the replication of successful interventions after a trial concludes. Similarly, in oncological interventions, variability in surgical methods may alter treatment effects, leading to inconsistent outcomes across groups.^5^

The CONSORT statement for Randomized Trials of Nonpharmacologic Treatments (NPT) is an evidence-based guideline designed to improve research clarity. However, its latest update highlights ongoing issues with adherence to the NPT extension for RCTs.^6^ New surgical procedures are recommended to be objectively measured and standardized to ensure quality and consistency in trials. This is particularly crucial in explanatory trials, where treatment efficacy is assessed in a controlled setting with a few expert surgeons, while pragmatic trials evaluate its effectiveness in real-world clinical practice. The importance of SQA in these so-called pragmatic trials lies in assessing and quantifying the degree of heterogeneity in operative importance. SQA leads to better adherence to the optimized standardized procedure if performed by the trained participants. Furthermore, by assessing this greater range in operative performance, investigators can evaluate the true content validity of SQA tools. Incorporating evidence-based SQA criteria into CONSORT guidelines could provide a standardized approach for trial design and reporting.

Since outcomes of RCTs have major impact upon surgical practice worldwide and are the fundaments of treatment protocols and (inter)national guidelines there is a need improve the implementation by adding SQA as a structural element of surgical trials. Further progress in surgical research requires structured trial methodology training, adherence to guidelines, and enhanced research infrastructure.^7^, ^8^, ^9^

The most effective methods to standardize surgery and to minimize variability in performances and corresponding outcomes remain unclear. A consensus on the essential methods of SQA could form the foundation for a widely accepted evidence-based and structured SQA checklist for surgical RCTs. Therefore, the primary objective of this Delphi survey was to establish expert consensus on what methods of surgical quality assurance which methods of quality assessment are most associated with reliable trial results due to minimal variation in procedures and to produce a universal checklist with key elements of surgical standardization.

## Methods

### Design

A 4-round modified Delphi consensus process was used to develop a SQA-checklist for RCTs in GI surgery.^10^^,^^11^ The process synthesized the opinions of surgeons conducting landmark RCTs and trial methodologists into a collective agreement on key factors for inclusion on the SQA-checklist.

Three authors (DvdA, SRM, SSG) served as the Delphi facilitators, responsible for preparing the questionnaires, sending invitations, monitoring responses, issuing reminders, and collecting, analyzing, and processing responses for subsequent rounds. The SQA-study group, comprising the authors, with expertise in clinical trials, SQA, and Delphi methodology coordinated the study and oversaw the design, implementation, and analysis of all Delphi rounds.

**Fig. 1:**
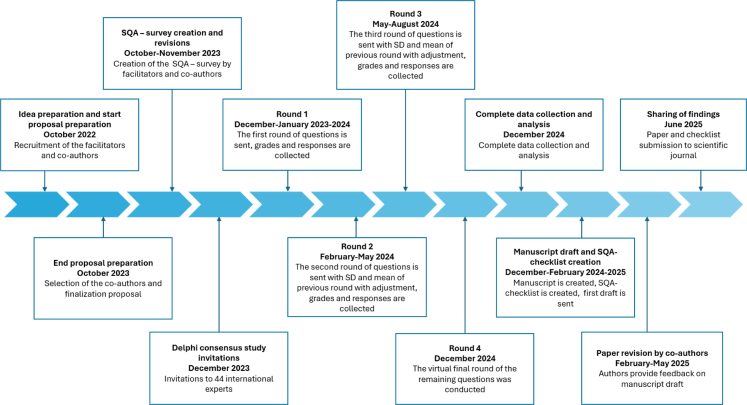
Workflow of the Delphi consensus study. Abbreviations: SQA, Surgical Quality Assurance; SD, standard deviation.

The primary design of and items included in the checklist were based on literature reviews and our systematic review.^2^^,^^5^^,^^12^ This review aimed to identify key SQA methods in RCTs to minimize bias in trial outcomes, categorized into three domains: standardization of surgical techniques, credentialing and monitoring of surgical performances.^5^ An updated version of this systematic review, reflecting a decade of growing attention to SQA, has recently been submitted. During each Delphi round, experts were asked to grade each checklist item using a 5-point Likert scale (1–5, strongly unimportant to very important). There was an option for free-text responses after each question, as well as an additional free-text section for general remarks at the end of each round. Ratings of 4 (‘important) and 5 (‘very important’) were combined and given equal weight in determining consensus for inclusion. Items rated ≥4 by at least 80% of experts were included in the final SQA checklist. Items rated ≥4 by 40–80% of experts proceeded to the next round, while items rated ≥4 by less than 40% of experts, or items with a mean lower than 3 were excluded, grounded on commonly accepted Delphi consensus standards.^13^ Based on the free-text responses new topics or textual modifications (highlighted in bold) suggested by the Delphi-experts were added. In each subsequent round, mean and standard deviation (SD) from the previous round were shared. Consensus was achieved after four rounds: three quantitative surveys followed by a virtual meeting. The online questionnaire was generated using Qualtrics software, version January 2024 (Qualtrics, Provo, UT) to collect the data and comments. The workflow is shown in Fig. 1.

### Participants

Forty-four international experts, including clinicians and trial methodologists were invited. Participants were invited on account of their expertise as surgeon, clinician and/or researcher, with experience in landmark RCTs. These experts were approached via email, where the study's objectives were outlined. Invited experts were provided with 4 weeks respond time to participate. A maximum of four reminder notifications were issued during the rounds. No incentives or compensation was provided. Experts were acknowledged as contributors in the DELPHI-study group.

### Ethics

As this was a Delphi consensus study, formal review or approval by an ethics committee was not required. Informed consent was obtained from all experts.

### Four Delphi rounds

The first Delphi round was conducted from December 2023 to January 2024. Experts were asked to rate 24 items in three categories using a 5-int Likert scale: (1–5, strongly unimportant to very important). In addition to each multiple-choice item, there were a free text field to provide a short explanation to support a rating. Respondents were also able to add new methods to the list that they considered potentially important for discussion in the subsequent round or provide additional commentary in a free-text format.

The second Delphi round took place from February 2024 to May 2024. Responses and comments from the first round, were collected and analyzed. Based on the ratings and comments, items on the survey were modified, deleted or supplemented, if applicable. The second round consisted out of 22 questions. Items on which consensus was reached in the first round were excluded.

The third Delphi round took place from May 2024 to August 2024. The survey composed of items for which consensus was not achieved in round one or two, and of any new items suggested in previous rounds. Again, for each item the mean score given by the overall group in the previous round was displayed, as well as the rating the experts gave in the previous round. Participants were able to revise their score with the additional knowledge of the results of our review.

For the final consensus round, a virtual meeting was organized in December 2024. From five questions, the last two resulting non-consensus checklist items from round 3 were resolved by discussion among the attendees. The final research questions deemed ≥4 by at least 80% of experts were formally approved.

### Statistics

Descriptive statistics were applied to summarize expert responses across all rounds. In each round, the mean and SD were calculated. This process followed established Delphi methodology. All analyses were performed using IBM SPSS Statistics, Version 28.0 (Chicago, USA).

### Role of the funding source

No funding was received for this study.

## Results

**Fig. 2:**
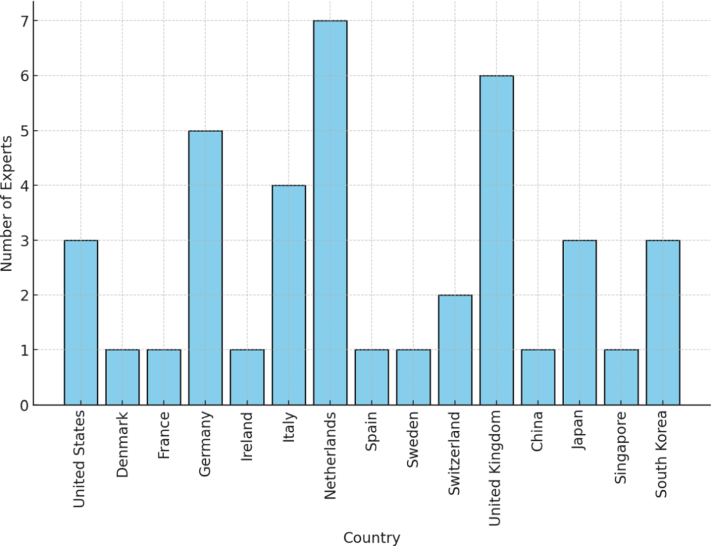
Geographical distribution of participants by country in round 3 (n = 40).

Forty four experts were invited, all with experience in one or more landmark RCT in GI surgery. Round 1 was completed by 43/44 experts (98% response rate). The participants included 39 surgeons and 4 trial methodologists from 15 countries across three continents (Asia, North America, and Europe) (Fig. 2). In this first round, experts evaluated 24 SQA topics categorized into three groups: credentialing (7 topics), standardization of surgical techniques (10 topics), and monitoring of surgical performance (7 topics). Following the assessment, one topic met the criteria for inclusion in the final checklist, while three topics were removed. Participants provided 199 topic-specific remarks, primarily addressing the overall study design, along with 8 general comments. These inputs were analyzed and used to refine topics for Round 2, ensuring greater clarity on the study design and research question. Also, one extra topic was added (see Supplementary File 3).

Round 2 was completed by 41 of the 43 experts from Round 1 (95% response rate). In this round, 21 SQA topics were evaluated, consisting of 7 topics in credentialing, 9 in standardization, and 5 in monitoring. One new topic was added to the credentialing category based on feedback from Round 1. Seven topics were deemed eligible for the final checklist, while two topics were removed from the Delphi questionnaire. The experts submitted 82 topic-specific comments and 5 general remarks, which informed refinements for Round 3, and one topic was added (see Supplementary File 3).

Round 3 was completed by 40 of the 41 experts from Round 2 (98% response rate). This round focused on 8 SQA topics, including 5 in credentialing, none in standardization, and 3 in monitoring. One additional topic was introduced in the credentialing category. Participants provided 62 topic-related remarks and 4 additional suggestions. At this stage, three topics were included in the final checklist, while the remaining five topics were discussed in the online meeting (see Supplementary File 3).

Nineteen SQA experts participated in the interactive discussion on the final five topics. They discussed and voted using the same methodology as in previous rounds, leading to the development of a framework for SQA in RCTs in GI surgery. After four rounds of consensus-building, the process concluded with agreement on 13 key items (Table 1). Each checked box was awarded 1 point, with a maximum possible score of 13 points. Based on the total score, quality thresholds can be categorized as follows: scores from 0 to 4 will indicate poor quality control, scores from 5 to 9 will indicate moderate quality control, and scores from 10 to 13 will indicate strong quality control (see Supplementary File 3). The removed questions are listed in Table 2.

**Table 1:** Framework for Surgical Quality Assurance in randomized controlled trials in gastrointestinal surgery.

**Table 2:** Overview of removed questions during Delphi consensus process.

	Scoring (total ≥4 (%); mean ± SD)			
	Round 1	Round 2	Round 3	Round 4
**Part A credentialing**				
How important is the assessment of operative reports/written surgical details, to use as a gatekeeper for trial entry?	34.8; 3.1 ± 1.0^a^	36.6; 3.4 ± 0.9	–	–
How important is, if already established nationally, contribution of data to national audits, to use as a gatekeeper for trial entry?	48.5; 3.4 ± 0.9	48.8; 3.4 ± 0.9	47.5; 3.4 ± 0.9	27.7; 3.0 ± 0.9
How important is video assessment, to use as a gatekeeper for trial entry?	48.8; 3.6 ± 0.9	75.7; 3.8 ± 0.7	62.5; 3.6 ± 0.8	50.0; 3.2 ± 1.1
How important is live operating theater assessment, to use as a gatekeeper for trial entry?	23.3; 2.8 ± 1.0	–	–	–
How important is standardized infrastructure in the hospital (e.g. 24/7 endoscopy interventional radiology, and other diagnostics) to use as a gatekeeper for trial entry?	–	–	60.0; 3.5 ± 0.9	29.4; 2.9 ± 1.1
**Part B standardization of surgical techniques**				
How important is standardization of materials and equipment, as a core component of the surgical procedure under investigation (e.g. staplers), standardize surgical techniques?	18.6; 2.7 ± 0.9^a^	11.2; 2.6 ± 0.9	–	–
How important is standardization of the anastomotic techniques as a core component of the surgical procedure under investigation (e.g. E–S or S–S, circular, linear or handsewn) to standardize surgical techniques?	37.2; 3.3 ± 1.0^a^	36.6; 3.5 ± 0.9	–	–
How important is pretrial education through live demonstration, to standardize surgical techniques?	39.5; 3.0 ± 1.0^a^	31.7; 3.2 ± 0.9	–	–
How important is proctoring surgeons with extensive experience in performing current established techniques, in the new intervention or technique under investigation, before trial entry?	27.9; 3.1 ± 1.0	29.3; 3.1 ± 0.9	–	–
**Part C monitoring of surgical performances**				
How important is video assessment of 100% of the cases, to monitor surgical performances?	21.0; 2.6 ± 1.2	–	–	–
How important is assessment of the complete operation video, to monitor surgical performances?	27.9; 2.9 ± 0.9	–	–	–
How important is monitoring using (national) audit data, if already established, to monitor surgical performances?	51.9; 3.2 ± 1.0	26.8; 3.2 ± 0.7	–	–
How important is video assessment of random selected procedures, to monitor surgical performances?	67.4; 3.8 ± 0.9	63.5; 3.6 ± 0.8	55.0; 3.5 ± 0.9	29.4; 2.9 ± 1.1

*Abbreviations: e.g.; for example,* E-S; end-to-side, S-S; side-to-side, SD; standard deviation.

a Proceeded to subsequent round in the questionnaire due to unclear questioning and remarks in free-text; - question was not included in this round

## Discussion

This International Delphi study investigated surgical trial experts’ opinion on several methods of SQA and developed a framework for SQA consisting of 13 items for standardization of surgical techniques, credentialing and monitoring of surgical performance in RCTs in gastrointestinal surgery. Credentialing of surgeons and centers, standardization of surgical techniques, and monitoring of surgical performance, enhances quality of surgical trials and improves both internal and external validity of study results. This framework will aid in building a strong SQA program and in evaluating SQA programs in published RCTs.

A systematic review highlighted that standardization of surgical techniques by SQA reduces variation in outcomes.^5^ The reliability of multicenter RCTs in surgical research is challenged by unmeasured variations within and between studies, despite their status as the highest level of evidence and foundation for practice-changing meta-analyses. Conventional statistical tests fail to capture this variability, underscoring the need for cautious interpretation of outcomes. To address this, implementing a Surgical Quality Assurance checklist can help standardize GI surgical procedures, reducing variability across surgeons, centers, and countries in RCTs. Moreover, SQA measures have been shown to reduce the risk of performance bias in surgical GI RCTs after successfully implementing quality assurance mechanisms on laparoscopic colorectal surgery to mitigate this bias, offering a potential framework for standardizing surgical quality in future trials.^12^

Some SQA topics received strong consensus in the four rounds but did not reach the final SQA framework. For example, the use of video recordings for surgical quality analysis seems an objective way to evaluate surgical quality, though they remain unfeasible in open surgery. Also, in minimally invasive procedures challenges remain. Security of privacy with video storage, storing capacity of recordings, costly processing of the videos, time consuming and costly assessment by human assessors are factors that were mentioned by the specialists as reason not to add video assessment to the checklist. However, successful examples of video assessment, both for credentialling as well as for monitoring during the trial exist, and videos can also be used to show the standardized technique. In the COLOR III trial, a quality assurance protocol including, histopathology reassessment, standardized surgical techniques, and surgical performance monitoring ensured consistent trial entry criteria and supported the development of reliable and valid clinical assessment tools. As part of this process, three videos were submitted and assessed by two independent reviewers before trial entry.^14^^,^^15^ Furthermore, the KLASS-02 trial also employed surgeon quality control, marking a milestone in the standardization of surgical procedures for clinical trials in gastrectomy.^16^ The TIGER-SQA study group is developing an artificial intelligence-based SQA tool to assess the completeness of lymphadenectomy and account for its variability in the TIGER study results. The tool utilizes short video clips, making the assessment process less labor-intensive.^17^ This reflects a broader shift toward digitalization and artificial intelligence development, which could potentially be applied to these videos and, could also be extended to other types of resections in the future.^17^, ^18^, ^19^

Moreover, emerging trial designs like stepped-wedge RCTs (SW-RCTs), and registry-based RCTs (RB-RCTs) and trials-within-cohorts (TwiCs) could also present solutions. For example, registry-based RCTs can incorporate real-world performance data, stepped-wedge designs enable phased implementation of SQA measures across centers, and TwiCs designs allow quality monitoring to be integrated within existing observational cohorts. However, they also introduce specific methodological and practical limitations. For example, stepped-wedge designs may be vulnerable to time-related confounding, complex statistical analysis requirements, and logistical challenges in coordinating staggered intervention rollouts. Therefore, while these innovative designs hold promise, their application requires careful consideration to ensure they enhance rather than compromise trial quality.^20^^,^^21^ The use of alternative trial designs in surgical research might contribute to quality in surgical trails. When applied appropriately, these innovative designs help address key quality challenges.^22^

The main strength of this study is its unique contribution and framework to enhance quality in surgical trials. However, the results of this study should be interpreted considering some limitations. First, our selection of SQA topics was based on existing literature, most of which originates from Europe and North America. However, in recruiting experts, we deliberately sought diverse representation across geographical regions and specialties, which helps to mitigate this initial limitation and broaden the relevance of our findings. Second, the ambiguity of answer options in round one, causing potential misinterpretation related to study design and trial-specific meanings. To address this, unclear items were carried forward to the next round despite that they failed to meet the inclusion threshold. This underscores the need for precise wording in Delphi studies to ensure accurate interpretation and expert consensus. Third, there was a minimal attrition in participation across the three Delphi rounds, a common challenge in Delphi studies involving multiple survey phases. Attrition between rounds 3 and 4 was anticipated given the transition from flexible online surveys to a scheduled online meeting at a fixed time. Nevertheless, overall retention remained high, supporting the continuity and methodological robustness of the consensus process. To ensure minimal participant loss we sent surveys to all initial participants and issued four reminder emails to encourage engagement.

Fourth, while the first three Delphi rounds were conducted anonymously, the final consensus round took place during a virtual meeting to allow in-depth discussion of remaining topics. This format inherently limited anonymity, which may have introduced bias despite the use of anonymous voting mechanisms. For this reason, it is crucial to mandate only the essential requirements, keeping them minimal and practical rather than idealistic, to ensure feasibility in real-world implementation. Fifth limitation includes the challenge of standardization with real-world applicability. While video-based evaluation, as described earlier, can provide objective insights into technical performance, we recognize that implementation may be resource-intensive and not feasible for all centers in the world (e.g. low- and middle-income countries) or trial designs. Notably, successful large-scale implementation of video-based SQA has been demonstrated in trials such as the COLOR III trial in colorectal surgery and the Korean KLASS trials in gastric surgery.^15^^,^^16^

While standardization enhances internal validity by ensuring consistency and reducing variability in surgical trials, some argue it may limit generalizability by creating an overly controlled study environment that does not fully reflect real-world clinical practice. A counterargument is the extent to which trials should regulate surgical quality. While trials impose strict controls, real-world implementation varies with surgeon expertise. Effective interventions must be robust enough for the average surgeon unless specialized training is incorporated into standard practice. Therefore, striking a balance between standardization and adaptability is essential to improve the applicability of surgical RCTs, ensuring that findings are both methodologically robust and clinically relevant.^23^, ^24^, ^25^

To support the framework's utility and generalizability, we propose a validation plan involving retrospective application of the checklist to landmark RCTs in gastrointestinal surgery by multiple independent reviewers. This will allow assessment of interrater agreement, scoring feasibility, threshold adjustments, and alignment with reported trial outcomes. It may also reveal which items are less practical or offer limited added value, informing future refinement of the checklist. Additionally, the process may highlight checklist items that are consistently difficult to score or contribute little to overall quality differentiation, thereby informing targeted refinement and optimization of the checklist.

In conclusion, in this study, international experts with expertise in landmark RCTs developed a framework for SQA consisting of 13 key items regarding standardization of surgical techniques, credentialing, and monitoring of surgical performance in RCTs in GI surgery. Incorporating evidence-based SQA could enhance both reliability and generalizability in GI surgical RCTs. This framework for SQA is expected to be widely utilized to enhance the quality of surgical trials and improve both their internal and external validity of upcoming study results. For future research, the checklist's applicability could be validated by assessing its relevance across a diverse set of published RCTs.

## Contributors

All authors contributed to the design and conceptualization of the study and the development of the Delphi questionnaire. D.C. van der Aa, S.R. Markar and S.S. Gisbertz coordinated the Delphi rounds and expert workshop. M.G. Besselink, J.B. Tuynman, N.S. Blencowe, G.B. Hanna, M.I. van Berge Henegouwen and H.J. Bonjer provided clinical oversight, methodological guidance and facilitated international collaboration. Data curation was performed by D.C. van der Aa and S.P.G. Henckens. Quantitative analyses were carried out by D.C. van der Aa, S.S. Gisbertz and S.R. Markar. All authors contributed to the interpretation of findings. The manuscript, including all tables and figures, was drafted by D.C. van der Aa and S.P.G. Henckens. All authors had full access to the data and critically revised the manuscript for intellectual content, and all authors read and approved the final version of the manuscript. S.P.G. Henckens, S.S. Gisbertz, and S.R. Markar have verified the underlying data. S.S. Gisbertz and S.R. Markar jointly supervised the project on behalf of the SQA-study group and Delphi-study group.

## Data sharing statement

Data supporting the findings of this study are available from the corresponding author upon reasonable request.

## Declaration of interests

N.S. Blencowe declares winning the MRC Clinician Scientist award, the remaining authors claim no conflicts of interests and no disclosures.
