# Supplementary material for: A framework for Surgical Quality Assurance (SQA) in randomized controlled trials in gastrointestinal surgery: an international Delphi consensus study
Source: eClinicalMedicine. 2025 Nov 13;90:103634. doi: 10.1016/j.eclinm.2025.103634 (PMC12661345; doi:10.1016/j.eclinm.2025.103634)
Supplement: Supplementary File 1 [file mmc1.docx]

**SUPPLEMENTARY FILE 1 – DELPHI-STUDY GROUP**

| **Title** | **Name** | **City** | **Country** | **Expertise** |
| --- | --- | --- | --- | --- |
| prof. dr. | Jonathan Cook | Oxford | United Kingdom | Methodologist |
| prof. dr. | Marion Campbell | Aberdeen | United Kingdom | Methodologist |
| prof. dr. | Barnaby Reeves | Bristol | United Kingdom | Methodologist |
| prof. dr. | Marcel Dijkgraaf | Amsterdam | Netherlands | Methodologist |
| prof. dr. | Willem Bemelman | Amsterdam | Netherlands | Colorectal Surgeon |
| dr. | Roel Hompes | Amsterdam | Netherlands | Colorectal Surgeon |
| prof. dr. | Stefan Benz | Böblingen | Germany | Colorectal Surgeon |
| prof. dr. | Antonino Spinelli | Milano | Italy | Colorectal Surgeon |
| prof. dr. | Steven Wexner | Cleveland | United States | Colorectal Surgeon |
| prof. dr. | Frederic Ris | Geneva | Switzerland | Colorectal Surgeon |
| dr. | Issam Al-Najami | Odense | Denmark | Colorectal Surgeon |
| prof. dr. | Michel Adamina | Sankt Gallen | Switzerland | Colorectal Surgeon |
| dr. | Cherylin Fu Wan Pei | Singapore | Singapore | Colorectal Surgeon |
| prof. dr. | Maasaki Ito | Chiba | Japan | Colorectal Surgeon |
| dr. | Rutger-Jan Swijnenburg | Amsterdam | Netherlands | HPB Surgeon |
| prof. dr. | Thilo Hackert | Hamburg | Germany | HPB Surgeon |
| prof. dr. | Tobias Keck | Lubeck | Germany | HPB Surgeon |
| prof. dr. | Mo Abu Hilal | Brescia | Italy | HPB Surgeon |
| prof. dr. | Jin-Young Jang | Seoul | South Korea | HPB Surgeon |
| prof. dr. | Henry Pitt | New Jersey | United States | HPB Surgeon |
| prof. dr. | Shishir Maithel | Atlanta | United States | HPB Surgeon |
| prof. dr. | Chris Halloran | Liverpool | United Kingdom | HPB Surgeon |
| prof. dr. | Rong Liu | Beijing | China | HPB Surgeon |
| prof. dr. | Bas Groot Koerkamp | Rotterdam | Netherlands | HPB Surgeon |
| prof. dr. | Jelle Ruurda | Utrecht | Netherlands | UGI Surgeon |
| dr. | Johanna van Sandick | Amsterdam | Netherlands | UGI Surgeon |
| prof. dr. | Guillaume Piessen | Lille | France | UGI Surgeon |
| prof. dr. | Peter Grimminger | Mainz | Germany | UGI Surgeon |
| prof. dr. | Jane Blazeby | Bristol | United Kingdom | UGI Surgeon |
| prof. dr. | Magnus Nilsson | Stockholm | Sweden | UGI Surgeon |
| prof. dr. | John Reynolds | Dublin | Ireland | UGI Surgeon |
| prof. dr. | Young-Woo Kim | Goyang-Si | South-Korea | UGI Surgeon |
| prof. dr. | Woo Jin Hyung | Seoul | South-Korea | UGI Surgeon |
| prof. dr. | Mitsuru Sasako | Osaka | Japan | UGI Surgeon |
| prof. dr. | Christiane Bruns | Cologne | Germany | UGI Surgeon |
| prof. dr. | Ricardo Rosati | Milano | Italy | UGI Surgeon |
| prof. dr. | Nick Maynard | Oxford | United Kingdom | UGI Surgeon |
| prof. dr. | Carlo Castoro | Milaan | Italy | UGI Surgeon |
| dr. | Marcos Bruna | Valencia | Spain | UGI Surgeon |
| dr. | Satoru Matsuda | Tokyo | Japan | UGI Surgeon |
| *Abbreviations: HPB; hepatobiliary, UGI; upper gastrointestinal* | | | | |
