# Supplementary material for: A framework for Surgical Quality Assurance (SQA) in randomized controlled trials in gastrointestinal surgery: an international Delphi consensus study
Source: eClinicalMedicine. 2025 Nov 13;90:103634. doi: 10.1016/j.eclinm.2025.103634 (PMC12661345; doi:10.1016/j.eclinm.2025.103634)
Supplement: Supplementary File 2 [file mmc2.docx]

**SUPPLEMENTARY FILE 2**

**Table 1.** Overview accepted of Surgical Quality Assurance questions in gastrointestinal surgical randomized controlled trials

| **#** | **Part** | **Delphi Round** | **Question** |
| --- | --- | --- | --- |
| 1) | A | 2 | How important is a minimum case volume annually per center, to use as a gatekeeper for trial entry? |
| 2) | A | 2 | How important is a minimum case volume for surgeons (overall experience), to use as a gatekeeper for trial entry? |
| 3) | A | 3 | How important is a minimum case volume annually per surgeon, to use as a gatekeeper for trial entry? |
| 4) | A | 3 | How important is standardizing the reporting guidelines for surgical procedures (e.g. predefined items in the report) |
| 5) | B | 1 | How important is proctoring surgeons without experience in the new techniques, before trial entry? |
| 6) | B | 2 | How important is pretrial education through videos, to standardize surgical techniques? |
| 7) | B | 2 | How important is standardization of the surgical approach?  *Surgical standardization refers to the steps needed to maintain surgical approaches that do not differ between resections.* |
| 8) | B | 2 | How important is standardization of the extent of lymphadenectomy, to standardize surgical techniques in?  *Extent of lymphadenectomy refers to a similar approach used for a lymphadenectomy as part of the surgical procedure for cancer (e.g. D1 or D2 lymphadenectomy, two-field or three-field lymphadenectomy, specified lymph node stations including anatomical boundaries).* |
| 9) | B | 2 | How important is pretrial education through written information, to standardize surgical techniques? |
| 10) | B | 2 | How important is proctoring surgeons with limited experience in performing current established techniques, in the new intervention or technique under investigation before trial entry? |
| 11) | C | 2 | How important is periodically pathology assessment for cancer surgery, to monitor surgical performances? |
| 12) | C | 3 | How important is monitoring using Case Report Form (CRF) or patient file data (e.g. recording of complications), to monitor surgical performances? |
| 13) | C | 4 | How important is random selected video assessment of a specific phase of the operation (e.g. anastomosis or lymphadenectomy), to monitor surgical performances? |
| *Part A: Credentialing; part B: Standardization of surgical techniques; part C: Monitoring of surgical performances* | | | |
