# Supplementary material for: A framework for Surgical Quality Assurance (SQA) in randomized controlled trials in gastrointestinal surgery: an international Delphi consensus study
Source: eClinicalMedicine. 2025 Nov 13;90:103634. doi: 10.1016/j.eclinm.2025.103634 (PMC12661345; doi:10.1016/j.eclinm.2025.103634)
Supplement: Supplementary File 3 [file mmc3.docx]

**SUPPLEMENTARY FILE 3 - DELPHI COMMENTS PER ROUND**

| **Round 1: Credentialing** | |
| --- | --- |
| **Questions** | **Comments** |
| 1) How important is a minimum case annually per center, to use as a gatekeeper for trial entry? | - Low volume hospitals produce worse outcomes. Dilution of results might be the issue. For all other questions and checking the validity of RCTs registry studies are well suited. - Dedication and accordingly knowledge of persons and teams seem much more important to me. Most important, however, is whether the aim is to increase internal or external validity - In many Italian institutions, there are several divisions of surgery caring for the same disease. typically for CRS. So, it would not be significant to consider per center case load. - Hospital or surgeon's volume affect larger to morbidity and recovery from fatal morbidity. - Number of surgeries may help, if used in combination with some other criterion. But number of surgeries is useless if the surgeries done previously do not have 'fidelity' with the intervention to be evaluated. - Case volume is important for many, but not all, procedures - Depends on whether one thinks it is important to measure learning as an integral part of the trial. The is a school of thought that randomization should commence from first case - with adjustment made in the analysis - Local investigators should be beyond their learning curve as a minimum to avoid inclusion bias. - Importance of center volume depends on the complexity of perioperative care, rather than complexity of the surgery; very important for pancreatic and esophageal surgery, less important for many other surgical interventions. - The experience of the surgeons actually performing the operations is more crucial to quality. Each surgeon should have a sufficient caseload and should have one or two nearly equally experienced colleagues to discuss complex approaches, knowledge updates, and continuity of care. These colleagues do not necessarily have to be located at the same site or location as long as mutual enrichment and support can be guaranteed. So, a trial is in itself facilitating these conditions. - If it is felt this is needed as a site may not have adequate expertise. - It depends on the trial design, less important for pragmatic RCTs more important for efficacy ones |
| 2) How important is a minimum case annually per surgeon, to use as a gatekeeper for trial entry? | - Same as above. - See above - Not sufficient, but essential - The senior surgeon guarantees for the members of the team - Because the study should be the reflect of reality, a center effect could be enough - Not a helpful answer, I know, but this probably (a non-surgeon view) depends on the nature of the surgery - Related procedures should be considered - As above, depends on whether one thinks it is important to measure learning as an integral part of the trial. The is a school of thought that randomization should commence from first case - with adjustment made in the analysis - Randomized trials can be pragmatic, but should never be performed under suboptimal conditions that can easily be adjusted before the trial takes place. One should be eager to prevent false-negative trials because of sloppiness of trial conduct - As above depends on the trial design, less important for pragmatic RCTs more important for efficacy ones |
| 3) How important is a minimum case for surgeons (overall experience), to use as a gatekeeper for trial entry? | - Seems to over represent senior surgeons and thus prevent establishment of new techniques - While for established techniques has limited value, I believe that for NEW techniques, this is essential - As above - Again, I'm not a surgeon so my view may be misguided. But I suspect that some overall 'inexperienced' might still be able to carry out the intended intervention with good fidelity - and on pragmatic grounds such surgeons would be providing the surgeries in the future is the intervention is found to be effective/cost-effective. - Very experienced surgeons who have "slowed down" should be included - I think it depends on the condition investigated. I think it´s important to have so-called expert centers in phase 3 and 4 studies, but also very important not to discriminate on center volume if one wants to use research as a catalysator for introducing new methods. - High overall experience may allow to include surgeons with intermediate annual volume. - Depending on the trial this importance can vary... - Useful where specific expertise (that cannot be presumed to be available) is needed and more than one surgeon at a site will be involved. - Young surgeons in a high volume unit are usually supervised - It depends on the trial design, less important for pragmatic RCTs more important for efficacy ones |
| 4) How important is contribution of data to national audit, to use as a gatekeeper for trial entry? | - This is a clear proxy for dedication - It is important, but not all countries (on the contrary, very few overall) have this national audit systems established - Not every country has a national audit system - Presumably, this is being considered as a proxy for a surgeon who 'reflects' on her/his practice, etc. What is the evidence for this being a reasonable proxy? - Audits vary from country to country, some are mandatory while others are voluntary and others do not exist - Unlikely to provide data, if not participating in national audit. - The requirement of submission of data for audit is independent of the trial in my view - Some countries are not available - I find it important, but with a risk of excluding international collaboration that don’t have the infrastructure of national audit capability, but where data is enormous and of very good quality. - Differs a lot between countries: makes sense in Netherlands, not in most other countries. - This depends on national health service organization and audit. Hospital audit could be considered - It depends on the trial design, less important for pragmatic RCTs more important for efficacy ones |
| 5) How important is the assessment of operative reports, to use as a gatekeeper for trial entry? | - Seem a good proxy for procedure and anatomical knowledge - But only if this focuses on adherence to the intended intervention. - Ideally, operative reports should be "standardized" but this effort is not easy - Operation notes often not accurate enough to be used in a trial anyway, better to have video footage - Flagging as slightly unimportant as this may vary depending on the aim of the trial - whether explanatory or pragmatic - Not sure what you mean with operative reports. But I believe it will depend on the method of interest. I.e.. if you want to study CME surgery then it is important. - Safe to assume that expert teams have decent op notes. - The national audit should include information components enabling to assess the conduct of surgeries. - The problem is that they are not always valid, i.e. what surgeon says was done was not actually one |
| 6) How important is video assessment, to use as a gatekeeper for trial entry? | - Selection of best cases in videos might be a bias. - It is important if a selection for proficient surgeons is desired. It has to considered that this decreases external validity - very complex. in RCT with limited numbers can be considered as additional quality measure - Could be complicated to realize - Video doesn’t lie - certainly relevant to MIE and RAMIE, not easy for open cases - My instinct says important - but I'm not a surgeon and, as a methodologist, I appreciate that logistically this may be challenging, especially in a large pragmatic trial. - OK if MIS trial and videos are readily available but not for open surgery - Flagging as slightly unimportant as this may vary depending on the aim of the trial - whether explanatory or pragmatic. This would also make trials even more difficult to get off the ground - Operations should be standardized like age gender BMI for the case submitted - same as above - Not commonly done, but several studies have demonstrated that video assessment is a highly reliable method to measure quality. - The Hawthorne effect may trigger better performance during operations with surgeons aware of being video-assessed. This may not be representative for daily practice. - I think it is good if the operation is one conducive to this, but it's not needed per se. More important for early assessments versus assessment in the context of routine care (e.g. typical NHS Phase III trial). - It depends on the trial design, less important for pragmatic RCTs more important for efficacy ones |
| 7) How important is live operating theatre assessment, to use as a gatekeeper for trial entry? | - See above - I am not sure I understand this - Important but difficult to perform. - Could be discussed depending on the trial - A consideration for MIE and RAMIE - My understanding is that video assessment should be an adequate way to judge adherence to the intended intervention. - Again, not very practical. Training program would be better - I am not sure what this means - Flagging as slightly unimportant as this may vary depending on the aim of the trial - whether explanatory or pragmatic. This would also make trials even more difficult to get off the ground and hugely resource intensive. This is also not reflective of real life - Practically difficult. - I find it rather unreliable as performance and choice of strategy can be altered due to assessment live - Limited additional benefit to video assessment; nice, but doesn't justify the required travel and time commitment. Surgeons may send in their very very best video; this can be resolved by requiring multiple videos. - It should be offered to surgeons, when felt needed by surgeons themselves. Live assessment should be a facilitator, not a test condition. Per surgeon analysis of trial results. Can be used, after bias-correction for e.g. case mix, as feedback to participating surgeons for purposes of lifelong learning. - Critical in trials where standards matter in large scale pragmatic trials less so |

| **Round 1: Standardization of surgical technique** | |
| --- | --- |
| **Questions** | **Comments** |
| 8) How important is pretrial education through written information, to standardize surgical techniques? | - I'm sorry but I'm going to answer that I don't know for all of these. Doesn't this depend on the nature of the intended intervention (some things being evaluated may be minor changes to customary practice, some large)? - Worth doing - Needs to be documented - In my view what is important is that the level of standardization is agreed before the trial. The mode of delivery of that information is less relevant. Assuming that written information is the basic minimum threshold I have ticked that this is important - The surgical interventions under investigation should always be clear. It not only enhances the internal validity of the trial, but also allows other, non-participating surgeons to adopt the 'winning' approach during implementation. - As above - depends on overall design |
| 9) How important is pretrial education through videos, to standardize surgical techniques? | - I'm sorry but I'm going to answer that I don't know for all of these. Doesn't this depend on the nature of the intended intervention (some things being evaluated may be minor changes to customary practice, some large)? - Probably most useful - A single edited video can always me made to look good. Moderate value. - In my view what is important is that the level of standardization is agreed before the trial. The mode of delivery of that information is less relevant. Videos may be useful - Still relatively uncommon. But absolutely makes sense. - Depending on the already achieved level of standardization (or commonness) of the studied interventions. - As above - depends on overall design |
| 10) How important is pretrial education through live demonstration, to standardize surgical techniques? | - Depends on type of operation - I'm sorry but I'm going to answer that I don't know for all of these. Doesn't this depend on the nature of the intended intervention (some things being evaluated may be minor changes to customary practice, some large)? - May not be necessary if videos available - It would be hard to gather all participants and inconvenient. - Depends what this means (ie. live operating, cadaver training etc.) - In my view what is important is that the level of standardization is agreed before the trial. The mode of delivery of that information is less relevant. Live demonstration as a minimum would make trials hugely resource intensive and limit access - Same as above; too much time costs. - Although the previous comment also holds, the live demonstration may reveal assumed standards, where in fact more variation exists. Further, it can be a motivating, binding moment of doing the research as one consortium. - Recorded unedited videos work well - As above - depends on overall design |
| 11) How important is standardization of the surgical approach? Surgical standardization refers to the steps needed to maintain surgical approaches that do not differ between resections. | - Final results after the resection might be important (Photo standardization) not the way to reach this (individual) - depends on specific trial, generally the more a surgeon has to modify is original procedure the higher the complication rate or the longer the learning/adaption curve - I'm sorry but I'm going to answer that I don't know for all of these. Doesn't this depend on the nature of the intended intervention (some things being evaluated may be minor changes to customary practice, some large)? - Not all procedures require a resection - But you need to accept some variation, even when standardized - The core elements of the surgical approach should be standardized, however, additional elements may be identified as optional - There is no right or wrong in the surgical approach. - Important, but trade-off with allowing for some variations due to surgeon's preferences. - As above - depends on overall design |
| 12) How important is standardization of the extent of lymphadenectomy, to standardize surgical techniques? Extent of lymphadenectomy refers to a similar approach used for a lymphadenectomy as part of the surgical procedure (e.g. D1 or D2 lymphadenectomy, two-field or three-field lymphadenectomy, specified lymph node stations including anatomical boundaries). | - For most trials this seems essential. However, there are questions conceivable that might be addressed unrelated to lymphadenectomy - Depends on the outcome of the RCT - I'm sorry but I'm going to answer that I don't know for all of these. Doesn't this depend on the nature of the intended intervention (some things being evaluated may be minor changes to customary practice, some large)? - Lymphadenectomy not done with all procedures - Importance depends very much on the trial interventions and endpoints. - Depends on whether the approach is crucial to the "critical element" of the surgical procedure under investigation - Not sure about esophagectomy, but many studies have shown that (the extent of) lymphadenectomy and it's relation with OS is highly overrated. - I can't really assess I'm not a surgeon but this was one of the famous examples of a possible learning effect - As above - depends on overall design |
| 13) How important is standardization of the anastomotic techniques to standardize surgical techniques? (e.g. E-S or S-S, circular, linear or handsewn) | - Data show no differences between different techniques in various RCTS - Prefer pragmatic approach - See above - Depends on the nature of the trial - For some surgical technique this point is pretty important but for majority of procedures not so much. - Totally dependent on research question in trial - I'm sorry but I'm going to answer that I don't know for all of these. Doesn't this depend on the nature of the intended intervention (some things being evaluated may be minor changes to customary practice, some large)? - Anastomosis not performed with all procedures - Difficult since every surgeon has its own personal approach - It depends on the purpose of study. If it is related with anastomotic safety, it must be clarified. - Importance depends very much on the trial interventions and endpoints. - Anastomotic technique is not important, important is if surgeons can demonstrate acceptable outcomes with their technique - Depends on whether the approach is crucial to the "critical element" of the surgical procedure under investigation - Ot depends on the preference - Again, not sure for esophageal cancer. But for pancreatoduodenectomy, we have many RCTs with conflicting results. If the methods of the anastomosis is the objective of the trial, obviously, this needs to be standardized. Otherwise, accept variation between experts. - As above. - As above - depends on overall design. Depends on the research question :) |
| 14) How important is standardization of materials and equipment (e.g. staplers), standardize surgical techniques? | - Get (RCT ) data on difference first. - It all depends what kind of validity is aspired. If you standardize everything the results will only be valid for exactly this setting. So strictly speaking, the result may hardly be relevant for routine practice. - Not essential. Can be useful for specific secondary outcomes? - Depends on intervention and the extent to which these items are plausible co-interventions? - Important if materials utilized - Depends on whether the approach is crucial to the "critical element" of the surgical procedure under investigation - Mainly important if this is the primary aim of the trial. - I think standardize what the flexibility that will be allowed is important but that doesn't necessary or even usually need to be equated as a specific piece of equipment IMO. - Sorry to be so repetitive - all depends on overall study design |
| 15) How important is proctoring surgeons without experience in the new techniques, before trial entry? | - For new complex procedures like D3/CME clearly yes, if trial entry should not be discouraged at all - I'm sorry but I'm going to answer that I don't know for all of these. Doesn't this depend on the nature of the intended intervention (some things being evaluated may be minor changes to customary practice, some large)? - Importance depends very much on the trial interventions and endpoints. - The requirements for involvement in a trial should be the same as involvement in routine practice - I cannot imagine how anyone in the 21st century would just start doing a new procedure without proctoring. In fact, proper training in a fellowship is far superior to proctoring. - Only surgeons that completed the learning curve for a procedure should enter a trial in oncologic surgery nevertheless young surgeons can be included if an expert tutor is always present - Surgeons without experience should not participate (see first 2 questions). - As above |
| 16) How important is proctoring surgeons with limited experience in the new techniques, before trial entry? | - It should be assured that the surgeon is able to do the procedure - It depends on the technique and on the trial - Surely this depends on what else is done to train surgeons? - The requirements for involvement in a trial should be the same as involvement in routine practice - See above |
| 17) How important is proctoring surgeons with extensive experience in the new techniques, before trial entry? | - It should be assured that the surgeon has the same understanding of the procedure - Many experienced surgeons believe they do a certain technique while they don't follow the necessary steps - I'm sorry but I'm going to answer that I don't know for all of these. Doesn't this depend on the nature of the intended intervention (some things being evaluated may be minor changes to customary practice, some large)? - For standardization - The requirements for involvement in a trial should be the same as involvement in routine practice - In my opinion pretrial education is very important to standardize a procedure. Especially proctoring. - Silly question. They should probably be proctors, right? - As above |

| **Round 1: Monitoring** | |
| --- | --- |
| **Questions** | **Comments** |
| 18) How important is video assessment of random selected procedures, to monitor surgical performances? | - Especially in laparoscopic surgery. - A few example cases may suffice, junctional and mid esophageal case - Again, may depend on the intervention? - Video assessment would give strong internal validity as they would show whether the procedure was delivered as planned. However, this adds major resource which may make this infeasible in all settings (any guidance produced would have to be applicable in all settings including LMIC in my view) - Again the material is subject to selection bias, and I have never felt that the feedback is easily transferred to the operating theatre - Great idea. But video's "may got lost"....has been our experience for RPD in NL. - Depending on the extent that slight variation of the conduct of procedures may influence patient outcome. - Nice to have but not strictly necessary IMO. |
| 19) How important is video assessment of 100% of the cases, to monitor surgical performances? | - It might be ideal but not realistic. - I don't think 100% is necessary - but a minimum number (rather than a %) should be done, and the surgeon should not know in advance that a surgery is going to be chosen to be videoed. - But how is it possible to do ? AI? - It would be important but it takes so longtime to review all cases. - Importance depends very much on the trial interventions and endpoints. - It depends on the workload, it has to be realistic - If one had unlimited budget this might be desirable, especially if this was an explanatory trial. However, less so for a pragmatic trial - Whole video is practically not needed - Of course that is the best, but infeasible. Unless, some aspect of video assessment is the aim of the study. For example, OSATS of an anastomosis to predict outcomes. |
| 20) How important is assessment of the complete operation video, to monitor surgical performances? | - Depends on primary outcomes! - Only important to video the elements necessary to validate the fidelity of the surgery with respect to the intended intervention. - But how is it possible to do? AI? - Importance depends very much on the trial interventions and endpoints. - I think a selection of key parts of the operation would be sufficient. - Of course that is the best, but again infeasible. Should learn from experts with video assessment in bariatric surgery and more recently RPD. - The more experienced the surgeons are, the less one would (and should!) be in need of full monitoring of the surgical procedure. |
| 21) How important is video assessment of a specific phase of the operation (e.g. anastomosis or lymphadenectomy), to monitor surgical performances? | - Depends on the outcomes chosen for the RCT! - Logistical issues with video monitoring -- hard to orchestrate - I think this is very important - I have only answered #4 because I am not sure that the 'monitoring' of fidelity HAS to be done by video. - Importance depends very much on the trial interventions and endpoints. - Video assessment of the "critical surgical element" under study might be helpful. - But you first need to demonstrate that whatever you are assessing is worth assessing. For example, if (extent of) lymphadenectomy doesn't change OS (which is true for most cancers), than who care how it is done, right? - Depends upon what the comparison is, and how well established the procedure is (and what options are available). |
| 22) How important is monitoring using CRF or patient file data, to monitor surgical performances? | - Essential to rule out human errors - Ideally, a copy of this CRF should be included in the patient's medical record to ensure that the surgeon does not document the key steps differently in the CRF and the medical record (or not at all in the medical record). - Not sure I understand the question. What matters is whether the CRF fields are unambiguous, who is entering the data, and whether it can be verified. - Useful to address unexpected variation or to identify deviations from the standardized surgical protocol |
| 23) How important is monitoring using (national) audit data, to monitor surgical performances? | - where available - As above, not all countries are like NL - Possibly strongly unimportant. In my experience, national audits are very unlikely to move quickly enough to record the kinds of details needed for a trial. - When available - I guess this is not important if you are collecting the data in CRFs - Depends on the settings, national Danish data might be less informative than larger nations as disease burden is completely different. As an example my institute contribute with 70 rectal cancer resections for the national audit yearly, of around 450 cases, to me these numbers are too small to make sense in that context. - This is not about importance, but about considerably lower costs of your trial if you can use national audit data. However, just as above, depends on ambiguity of data fields, who is entering the data, etc. - Not always available, audit data can be quite selective depends attempts to be comprehensive. |
| 24) How important is pathology assessment, to monitor surgical performances? | - Depends on standardization in pathology - Surely depends on the intended intervention? - When resection of a malignancy is involved - Depends on the study. - Depends on the disease of interest, if it´s CME surgery I don’t think it´s important, but if you want to look at the impact of EMVI on outcomes it´s pivotal - In PDAC the pathology report mostly reflects commitment and expertise of the pathologist rather than the surgeon. Same holds for CCA. Probably also true for other cancers, but too a lesser extent. - Periodically by (unannounced) interval assessment may be sufficient. - Depends upon the procedures and content. Even this one step removed often from surgical aim so supportive but definitive. |
| Additional suggestions and remarks | - For the investigation of surgical techniques a baseline information of the surgeon and the institution in routine case should be available (e.g. audit data) also the standard technique prior to the trial for the procedure in question should be recorded. Experience of the surgeon and the department should be rated in broader terms like over-all procedures per year. This should, however, more be descriptive rather than a selection criterion. - Lots of these answers will vary on the specifics of a trial - This is not really about additional items - but I feel it is important to address the extent to which a trial should control surgical quality, since any intervention found to be effective/cost-effective will ultimately be performed without some of the controls on quality used in the trial - and by surgeons of varying quality. As a patient, i would want an intervention to be sufficiently robust for an average surgeon to perform it (unless very specialized training similar to the controls used for the trial were to be implemented in usual practice). - Perhaps be more specific with the questions - Need to get the questions right - I wonder whether the surgical field after resection can be helpful to assess, like the dissected branches of the SMV in CME surgery for example, or the intact mesorectum after TEM etc. I also think it´s important tp work on the dogma of regarding R1 as an indicator of surgical quality, but maybe regard it as a composite failure of not only surgery but the MDT as a whole, maybe an R1 is a result of failure of radiological staging, failure of oncological treatment or pathological assessment. So a retrospective review of MDT decision might have a place in SQA. - In wording the questions, important to specify what the objective of the study is. E.g., if you are introducing a new stapler for anastomoses, I wouldn't worry about lymphadenectomy. - quality analysis of the pathological examination and standards of different institutes...Maybe different definitions... - Addressing sustainability during the design of studies with respect to how surgical procedures should be performed and studies themselves should be conducted. Full monitoring may (!) not be as sustainable as possible. The more experienced the participating group of surgeons, the more sustainable one should design measures of quality assurance. - Educational hospitals and young surgeons in training, hospital audit |

| **Round 2: Credentialing** | |
| --- | --- |
| **Questions** | **Comments** |
| 1) How important is a minimum case annually per center, to use as a gatekeeper for trial entry? | - Mean is depending on complexity of procedure and access (MIS / Robotic ) . Might be higher. - Agree - If it is felt this is needed as a site may not have adequate expertise. Can be useful for certain types of studies. - For the sake of external validity those who take part in patient care should be able to take part in a trial otherwise the results cannot be transferred into routine practice |
| 2) How important is a minimum case annually per surgeon, to use as a gatekeeper for trial entry? | - Mean is depending on complexity of procedure and access (MIS / Robotic ) . Might be higher. - Hospital volume the more valuable criteria in my view - A sufficiently experienced center does not imply that all center surgeons are sufficiently experienced in performing operations in a trial context. - See previous |
| 3) How important is a minimum case for surgeons (overall experience), to use as a gatekeeper for trial entry? | - Mean is depending on complexity of procedure and access (MIS / Robotic ) . Might be higher. - Hospital volume the more valuable criteria in my view - Needs to be over his learning curve - Depending on the trial this importance can vary... - Useful where specific expertise (that cannot be presumed to be available) is needed and more than one surgeon at a site will be involved. More useful than site IMO but also can sometime be inferred if evaluating existing operation in widespread use. Getting info on this is beneficial for reporting but not always easy to reliably collect. - It really depends on whether you are taking the view of randomizing from first patient with active measurement of the learning curve or whether the perspective is that only "experienced" surgeons are to be in the trial - Depends on trial design - pragmatic or tightly controlled - so its trial dependent - For a randomized trial it isn’t important that the Surgeon is familiar technique that is tested. This may not apply for non-randomized settings like regression discontinuity trials |
| 4) How important is contribution of data to national audit, if already established nationally, to use as a gatekeeper for trial entry? | - I could not find any well-developed national audit still. - Mean is depending on complexity of procedure and access (MIS / Robotic ) . Might be higher. - It is important, in UK, Netherlands , Scandinavia (I think) and Ireland all esophageal centers must audit nationally, so it is important. - But is easy to check unit volume of procedures - Not practical for many surgical procedures. If a national audit exists it is good data is available. Not strictly necessary though as "gatekeeper" - Useful to understand the context around a trial but as above all is very trial dependent - Again, those who take part in patient care should be able to include patients in trails to assure external validity |
| 5) How important is the assessment of operative reports/written surgical details, to use as a gatekeeper for trial entry? | - It is too subjective. It cannot guarantee surgical quality. - Same comment - Logistics extremely difficult - Generally not needed but for very new/complex procedures could be worthwhile/necessary. - In theory this sounds good but the feasibility will be difficult - Often not consistent with what actually happened |
| 6) How important is standardizing the reporting guidelines for surgical procedures (e.g. predefined items in the report)  **NEW** | - Objective components and inclusion of essential element should be checked. - Excellent idea, a bit like complications, operative reports should be standardized and perhaps this is a further project this group can undertake. - Within trial important perhaps but should not be used for trial entry marker - All should speak the same |
| 7) How important is video assessment, to use as a gatekeeper for trial entry? | - I think it is good if the operation is one conducive to this. More important for early assessments versus assessment in the context of routine care (e.g. typical NHS Phase III trial). |

| **Round 2: Standardization of surgical techniques** | |
| --- | --- |
| **Questions** | **Comments** |
| 8) How important is pretrial education through written information, to standardize surgical techniques? | - Onsite observation is better because video can be manipulated. It can be other surgeon's performance. - Reasonable suggestion, perhaps 2 intitial videos, 2 or 3 stage - Or photographs which are easier - except for surgeons who were already assessed by video for the studied intervention on a previous occasion and was proven as qualified - My answers presume these are listed as requirements. Very happy for this to be an OPTION but it is not necessary in most situations to view videos of ops for all participating surgeons. - Could be useful, but again would not be essential in my view for inclusion - Unless a totally pragmatic trial design - this gives information about what really happened, not what surgeons reported to have happened - It is a very good tool to assess to assess surgical proficiency. However, the cutoff should only include a small proportion of expert, super expert surgeons. |
| 9) How important is pretrial education through videos, to standardize surgical techniques? | - Helpful so support - Useful but not generally necessary per se. - Depends on overall trial design |
| 10) How important is pretrial education through live demonstration, to standardize surgical techniques? | - As required - Useful but not generally necessary per se. - see recurring notes on trial design :) |
| 11) How important is standardization of the surgical approach?  Surgical standardization refers to the steps needed to maintain surgical approaches that do not differ between resections. | - This should be a check list, but many do a 3 stage and neck as opposed to high 2 stage, so discretion is required rather than a prescribed approach. - Standardize the options but not need to say necessarily do exactly this. Need to know surgeon were able to do under the protocol - See recurring notes on trial design :) |
| 12) How important is standardization of the extent of lymphadenectomy, to standardize surgical techniques in? Extent of lymphadenectomy refers to a similar approach used for a lymphadenectomy as part of the surgical procedure for cancer (e.g. D1 or D2 lymphadenectomy, two-field or three-field lymphadenectomy, specified lymph node stations including anatomical boundaries). | - Again, there is no gold standard so not relevant here I feel - Depends on the objective of the trial. If the aim is to investigate anastomosis, then it is a 5. - Depends upon the comparison - if this what is being compared (and the rest of op is unchanged then very important. If different procedures plans then flexibility here seems reasonable to me. - Unless the study is on technical details or anastomotic techniques - See recurring notes on trial design :) for example in a trial testing different anastomotic techniques this is critical, less important of evaluating robotic versus MIO |
| 13) How important is standardization of the anastomotic techniques as a core component of the surgical procedure under investigation (e.g. E-S or S-S, circular, linear or handsewn) to standardize surgical techniques? | - Because every centre has his own habits and need therefore to follow their habits but modify the technique for the study - In general but sometimes equipment is very linked to procedure. - Depends - if there is evidence that one form of equipment is better than another - if there is then yes, otherwise neutral - See recurring notes on trial design :) |
| 14) How important is standardization of materials and equipment, as a core component of the surgical procedure under investigation (e.g. staplers), standardize surgical techniques? | - Surgeons with limited experience SHALL not be participating, unless it would be a trial on learning curve of a given procedure |
| 16) How important is proctoring surgeons with limited experience in performing current established techniques, in the new intervention or technique under investigation, before trial entry? | - Yes if it is a new technique that is not standard or expert at the institution - Although there would be concern here - Useful and tends to happen automatically. So often doesn't need to be done for the trial. - See recurring notes on trial design :) and the procedure under evaluation - maybe they can be assisted by a more experienced surgeon |
| 17) How important is proctoring surgeons with extensive experience in performing current established techniques, in the new intervention or technique under investigation, before trial entry? | - Question needs to be more specific , the most experienced surgeons will need help in some novel techniques, so this question needs to be developed - What is the difference between a proctor and a surgeon with extensive experience? - But as above, there should be a clear collective understanding of how the procedure is to be undertaken for the trial - As above |

| **Round 2: Monitoring** | |
| --- | --- |
| **Questions** | **Comments** |
| 18) How important is video assessment of random selected procedures, to monitor surgical performances? | - Really depends on the trial, if surgery the key variable , or a new technique, then 4, if not then neutral. Again a specific scenario is required. - Nice but not necessary - Likely this would be a gold standard approach, but whether it should be mandated is debatable |
| 21) How important is video assessment of a specific phase of the operation (e.g. anastomosis or lymphadenectomy), to monitor surgical performances? | - Again, depends on the trial - The importance of the extent of lymphadenectomy is overrated in surgical oncology. Read Blake Cady. - More important if focused on key part but then again might as well video the whole op if you go to the bother of recording. - Likely this would be a gold standard approach, but whether it should be mandated is debatable - Depends on the research question |
| 22) How important is monitoring using CRF or patient file data, to monitor surgical performances? | - Please define CRF - Not required if Center has data management with integrity, have to trust - How does this work - Ask relevant Qs on OP and complications - Monitoring is mandatory by IRB/METC: so Delphi opinion irrelevant. |
| 23) How important is monitoring using (national) audit data, if already established, to monitor surgical performances? | - More as a control for provided data - Useful addition but not needed. |
| 24) How important is periodically pathology assessment for cancer surgery, to monitor surgical performances? | - Monitoring pathology assessment is mostly needed to monitor the performance of the pathologist. Would use same terminology (e.g. periodically) for all questions where you don’t want to do something for every single patient. - This would be part of routine care so should be getting done anyway in the background. - For cancer trials essential - But should be used as a measure of met performance not surgical performance only. I see a R1 as a failure of MDT could be due to wrong staging, poor downstaging and not only a surgical failure. |
| Additional suggestions and remarks | - Defining and categorization of intra-operative complications and reporting - Use national qualification programs when available - I would make a list of 10 published RCTs to which the guideline should be applicable. Then, check for each paper whether the questions are appropriate. Make sure that the selected RCT cover the entire field. - Pathological quality and standardization of definitions... - Standardized infrastructure e.g. endoscopy 24/7, interventional radiology 24/7, other diagnostics 24/7 |

| **Round 3: Credentialing** | |
| --- | --- |
| **Questions** | **Comments** |
| 1) How important is a minimum case annually per surgeon, to use as a gatekeeper for trial entry? | - Depends on the philosophy of the trial whether randomize from first case to characterize the learning within the trial or not - I am 'going with the flow' here. - Depends also on his previous experience and how many cases he has done - Learning curves are personal not only by institution. - Case volume per surgeon more important than institutional - Surgeons tend to inflate their figures and numbers to not equate to competence - Overall Experience is important for senior surgeons - Either total experience or minimum annual case load. This can be variable for very experienced surgeons with great cumulative experience and young surgeons - More specifically last 2 years average number of cases would be better. - If it is felt this is needed as a site may not have adequate expertise. Can be useful for certain types of studies. - This question is a trade-off between internal and external validity - Keep in mind that randomization itself may let the case number drop by 50% during the trial, which may result in surgeons not meeting the requirement for participation in the next study. |
| 2) How important is, if already established nationally, contribution of data to national audits, to use as a gatekeeper for trial entry? | - Most countries simply don't have nationwide audit - If to restrictive may introduce a limit in applicability of the results of the trial. Additionally those data can be influenced by the complexity of cases or the beginning of a learning curve - Of course this is desirable but I see no reason to assume this is a good proxy for adherence to the proposed trial. I'd want to examine the reasons why a potential surgeon participant was not participating in the national audit. - In most cases this is externally validated data- - can be used to determine denominator and cross reference for data points - The senior surgeon guarantees for the members of the team - Transparency is an indicator of standards of care - Data verification of centers is key - I value more important to have an institutional data base with prospectively collected data and regularly updated follow up. Participation in national databases and audit depends on local policies and could also be on a voluntary basis Obviously and if compulsory. |
| 3) How important is standardizing the reporting guidelines for surgical procedures (e.g. predefined items in the report) | - This will help with the reporting of trials in future - I'm not sure I understand the item correctly. My "5" response means that I think it is vital to define surgical procedures carefully with respect to a 'standard' number of items, and to report those items fully with respect to the definitions established at the outset. - As above - what surgeons say they do and actually do are different things - - Depending on the trial this importance can vary... - Otherwise too arbitrary. - Useful where specific expertise (that cannot be presumed to be available) is needed and more than one surgeon at a site will be involved. More useful than site IMO but also can sometime be inferred if evaluating existing operation in widespread use. Getting info on this is beneficial for reporting but not always easy to reliably collect. - This would be a good initiative that can be developed with EsoDATA - Reporting guidelines are important for meta-analyses of trials, real world data, and the mix of both. |
| 4) How important is video assessment, to use as a gatekeeper for trial entry? | - Photographic documentation also - Whilst ideal in theory, ensuring this level of assessment prior to trials will likely both affect the participation and will also increase the cost and the logistical requirements - Video assessment of what? Doing the procedures that the proposed trial plans to evaluate? I don't think that this is important for procedures that don't contribute to the trial. If feasible, I do think it is important to check at least a sample of procedures included in the trial per surgeon. - Not every country has a national audit system - It depends on the trial design, but unless very pragmatic design it is critical to demonstrate to other surgeons the standards of surgery - Depending on the study question... - Can be short video clips to show surgical field after the main procedure. after the - Not practical for many surgical procedures. If a national audit exists it is good data is available. Not strictly necessary though as "gatekeeper" - This is a worthy objective but logistics and GDPR complicate the issue. Only designated accredited esophageal centers should be included, where this is not done as in Checkmate 577 the overall outcomes may be poor as a direct result? - Very much dependent on the simplicity or complexity of the operation |
| 5) How important is standardized infrastructure in the hospital (e.g. 24/7 endoscopy interventional radiology, and other diagnostics) to use as a gatekeeper for trial entry? **NEW** | - Depending on the primary outcome. You don't need IR for margin-status as primary outcome. But infrastructure essential for 90-day mortality and failure to rescue. - Depends on the trial - Otherwise should not perform those kind of operations - I think this is a very odd question. Surely it depends on the nature of the question? - Data is clear on that - Depends on what the main outcome measures are for the trial - Context is critical to outcomes - so a quality marker of the center would be important I think - High volume units should guarantee the availability of these services - Generally not needed but for very new/complex procedures could be worthwhile/necessary. - Possibly 5, consistent with only using designated, accredited or centralized hospitals who by definition will have these structures in place - This is dependent on the research question and disease context. |
| **Round 3: Credentialing** | |
| **Questions** | **Comments** |
| 1) How important is a minimum case annually per surgeon, to use as a gatekeeper for trial entry? | - Depends on the philosophy of the trial whether randomize from first case to characterize the learning within the trial or not - I am 'going with the flow' here. - Depends also on his previous experience and how many cases he has done - Learning curves are personal not only by institution. - Case volume per surgeon more important than institutional - Surgeons tend to inflate their figures and numbers to not equate to competence - Overall Experience is important for senior surgeons - Either total experience or minimum annual case load. This can be variable for very experienced surgeons with great cumulative experience and young surgeons - More specifically last 2 years average number of cases would be better. - If it is felt this is needed as a site may not have adequate expertise. Can be useful for certain types of studies. - This question is a trade-off between internal and external validity - Keep in mind that randomization itself may let the case number drop by 50% during the trial, which may result in surgeons not meeting the requirement for participation in the next study. |
| 2) How important is, if already established nationally, contribution of data to national audits, to use as a gatekeeper for trial entry? | - Most countries simply don't have nationwide audit - If to restrictive may introduce a limit in applicability of the results of the trial. Additionally those data can be influenced by the complexity of cases or the beginning of a learning curve - Of course this is desirable but I see no reason to assume this is a good proxy for adherence to the proposed trial. I'd want to examine the reasons why a potential surgeon participant was not participating in the national audit. - In most cases this is externally validated data- - can be used to determine denominator and cross reference for data points - The senior surgeon guarantees for the members of the team - Transparency is an indicator of standards of care - Data verification of centers is key - I value more important to have an institutional data base with prospectively collected data and regularly updated follow up. Participation in national databases and audit depends on local policies and could also be on a voluntary basis Obviously and if compulsory. |
| 3) How important is standardizing the reporting guidelines for surgical procedures (e.g. predefined items in the report) | - This will help with the reporting of trials in future - I'm not sure I understand the item correctly. My "5" response means that I think it is vital to define surgical procedures carefully with respect to a 'standard' number of items, and to report those items fully with respect to the definitions established at the outset. - As above - what surgeons say they do and actually do are different things - - Depending on the trial this importance can vary... - Otherwise too arbitrary. - Useful where specific expertise (that cannot be presumed to be available) is needed and more than one surgeon at a site will be involved. More useful than site IMO but also can sometime be inferred if evaluating existing operation in widespread use. Getting info on this is beneficial for reporting but not always easy to reliably collect. - This would be a good initiative that can be developed with EsoDATA - Reporting guidelines are important for meta-analyses of trials, real world data, and the mix of both. |
| 4) How important is video assessment, to use as a gatekeeper for trial entry? | - Photographic documentation also - Whilst ideal in theory, ensuring this level of assessment prior to trials will likely both affect the participation and will also increase the cost and the logistical requirements - Video assessment of what? Doing the procedures that the proposed trial plans to evaluate? I don't think that this is important for procedures that don't contribute to the trial. If feasible, I do think it is important to check at least a sample of procedures included in the trial per surgeon. - Not every country has a national audit system - It depends on the trial design, but unless very pragmatic design it is critical to demonstrate to other surgeons the standards of surgery - Depending on the study question... - Can be short video clips to show surgical field after the main procedure. after the - Not practical for many surgical procedures. If a national audit exists it is good data is available. Not strictly necessary though as "gatekeeper" - This is a worthy objective but logistics and GDPR complicate the issue. Only designated accredited esophageal centers should be included, where this is not done as in Checkmate 577 the overall outcomes may be poor as a direct result? - Very much dependent on the simplicity or complexity of the operation |
| 5) How important is standardized infrastructure in the hospital (e.g. 24/7 endoscopy interventional radiology, and other diagnostics) to use as a gatekeeper for trial entry? **NEW** | - Depending on the primary outcome. You don't need IR for margin-status as primary outcome. But infrastructure essential for 90-day mortality and failure to rescue. - Depends on the trial - Otherwise should not perform those kind of operations - I think this is a very odd question. Surely it depends on the nature of the question? - Data is clear on that - Depends on what the main outcome measures are for the trial - Context is critical to outcomes - so a quality marker of the center would be important I think - High volume units should guarantee the availability of these services - Generally not needed but for very new/complex procedures could be worthwhile/necessary. - Possibly 5, consistent with only using designated, accredited or centralized hospitals who by definition will have these structures in place - This is dependent on the research question and disease context. |

| **Round 3: Monitoring** | |
| --- | --- |
| **Questions** | **Comments** |
| 6) How important is video assessment of random selected procedures, to monitor surgical performances? | - To me this is the ideal scenario but not necessarily one which can be introduced as a minimum standard routinely. - better to provide short video of the result of the dissection - If feasible. Ultimately, this is the only way to verify what is recorded on the CRFs. The problem is whether the surgeon knows in advance that the video has been selected. Ideally, all procedures would be videoed (not such a huge requirement now?) - and a random selection chosen for assessment. - See earlier comments about videos - Can be important but technically difficult to get the videos. - Interesting proposal but extremely difficult to implement. Minimum hospital volumes , reporting outcomes, accreditation etc. are the keys. It may be possible to pilot this in the Netherlands to demonstrate concept. |
| 7) How important is video assessment of a specific phase of the operation (e.g. anastomosis or lymphadenectomy), to monitor surgical performances? | - Again, depending on the primary (and secondary) outcomes. - To me this is the ideal scenario but not necessarily one which can be introduced as a minimum standard routinely. - See above. My answers relate to parts of the procedures defined by 'standard items' (as per question near the beginning of the survey) - Depends completely on the trial in question - As per earlier comments - Not against it, nor are my colleagues , but as above will be difficult to get buy in from surgeons I suspect, again a pilot study would be helpful. |
| 8) How important is monitoring using Case Report Form (CRF) or patient file data (e.g. recording of complications), to monitor surgical performances? | - Depending on outcomes - This can be achieved by everyone and will provide a more comprehensive approach to recording - In addition to any video assessment (above), assuming that video assessment (better) is not feasible to do for all procedures. - Our studies show that surgeons report one thing but do something different - videos provide the hard evidence of what was actually done - This will be in the RCT CRF as standard. |
| Additional suggestions and remarks | - Q9 - Photo alternative to video - May not have read intro in detail. But these judgments strongly depend on the outcome selection. - Surgical margin and dissected lymph nodes number in malignant diseases - Having at least two formally trained HPB/Oncology/MIS surgeons care for the patients - Perhaps a re-discussion on video assessment methodology would be helpful at this juncture |

| **Round 4: Remaining questions** | |
| --- | --- |
| **Questions** | **Comments** |
| 1) How important is, if already established nationally, contribution of data to national audits, to use as a gatekeeper for trial entry? | - I can see reasons why this should, and should not, be important. So I don't think on balance that it is a good "item" for SQA. |
| 2) How important is video assessment, to use as a gatekeeper for trial entry? | - In my opinion impossible to standardize, we all have good videos and bad videos, we all know which videos we share, and are they then representative? - Also the issue is whether it is "essential" or nicer to have - if "essential" in , if nicer to have then n - Agree with Christiane entirely.  If want to be pragmatic and have wide applicability, need to include everyone.  you can collect path data (margin, LN yield, etc.) as a surrogate for quality of surgery - Another side of it is how to evaluate the videos this is also prone to subjective assessment - I agree with Johanna, problem of cost  and of time.... so ideally yes, realistically for the moment no. We are about to start a trial in France and i do not know how we will manage it from the realistic practical point of view |
| 3) How important is standardized infrastructure in the hospital (e.g. 24/7 endoscopy interventional radiology, and other diagnostics) to use as a gatekeeper for trial entry? | - None, clearly not to include in new checklist. |
| 4) How important is video assessment of random selected procedures, to monitor surgical performances? | - We need to be really careful about what we mandate MUST happen - in my view this should be a very minimal set of things (and should not include things that we'd like in an ideal world) - But now I think you will have a problem defining what you mean a trial of a "technical" question. - So, we may specify the question according to the nature of the study. - So for this question: if the aim is to investigate a specific surgical technique - For explanatory trials |
| 4) How important is video assessment of a specific phase of the operation (e.g. anastomosis or lymphadenectomy), to monitor surgical performances? | - Same remark as before if the aim is to evaluate lymphadenectomy of left paratracheal nodes such as Robot vs. TMIE yes otherwise no - Interested in knowing who will pick up randomly? Again a difficult task to complete logistically. - Additional change to question will be the adding of: RANDOMLY selected |
